# Supplementary figures and images for: Crowdsourced identification of multi-target kinase inhibitors for RET- and TAU- based disease: The Multi-Targeting Drug DREAM Challenge
Source: PLoS Comput Biol. 2021 Sep 14;17(9):e1009302. doi: 10.1371/journal.pcbi.1009302 (PMC8483411; doi:10.1371/journal.pcbi.1009302)

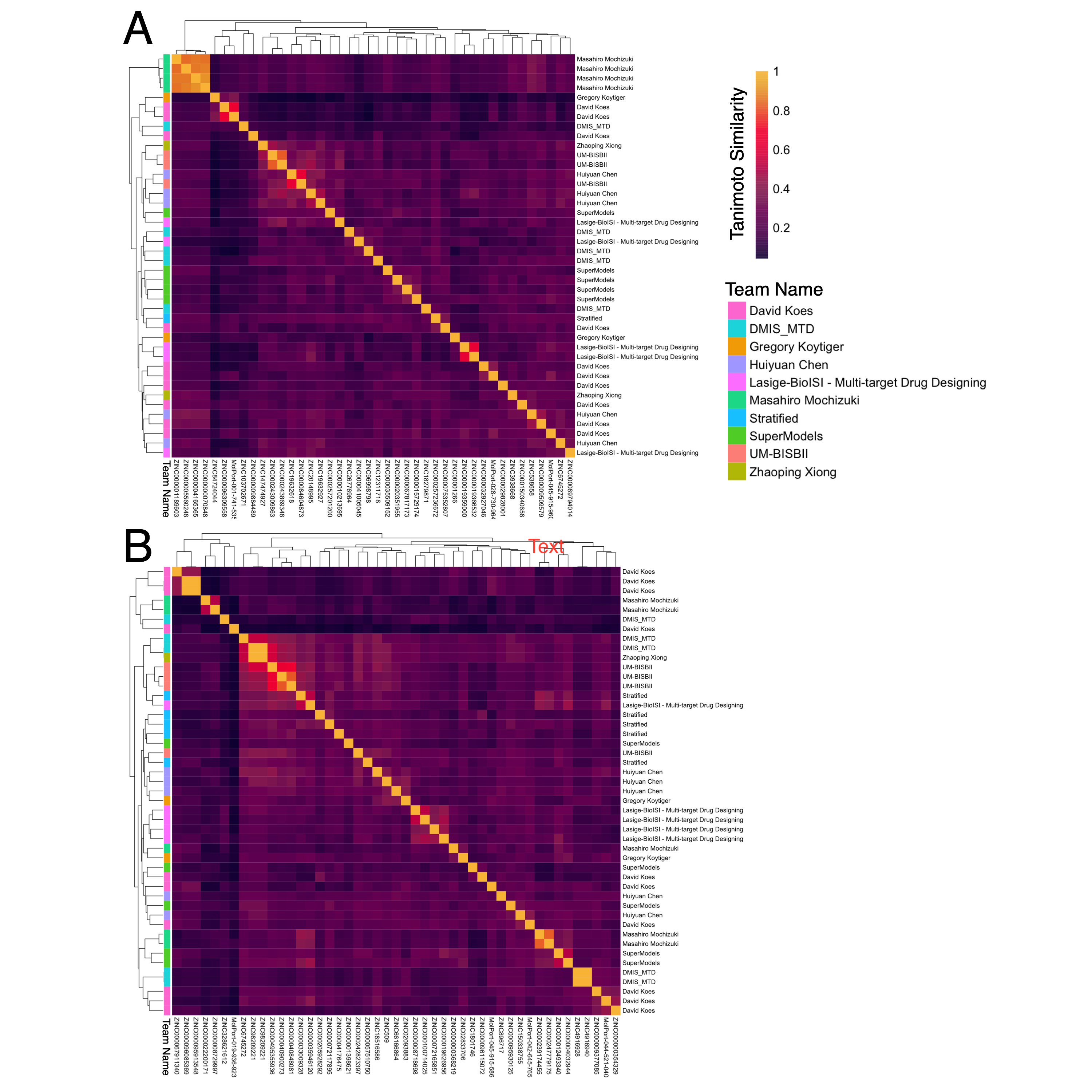

Supplement: S1 Fig — (TIFF) [file pcbi.1009302.s002.tiff]

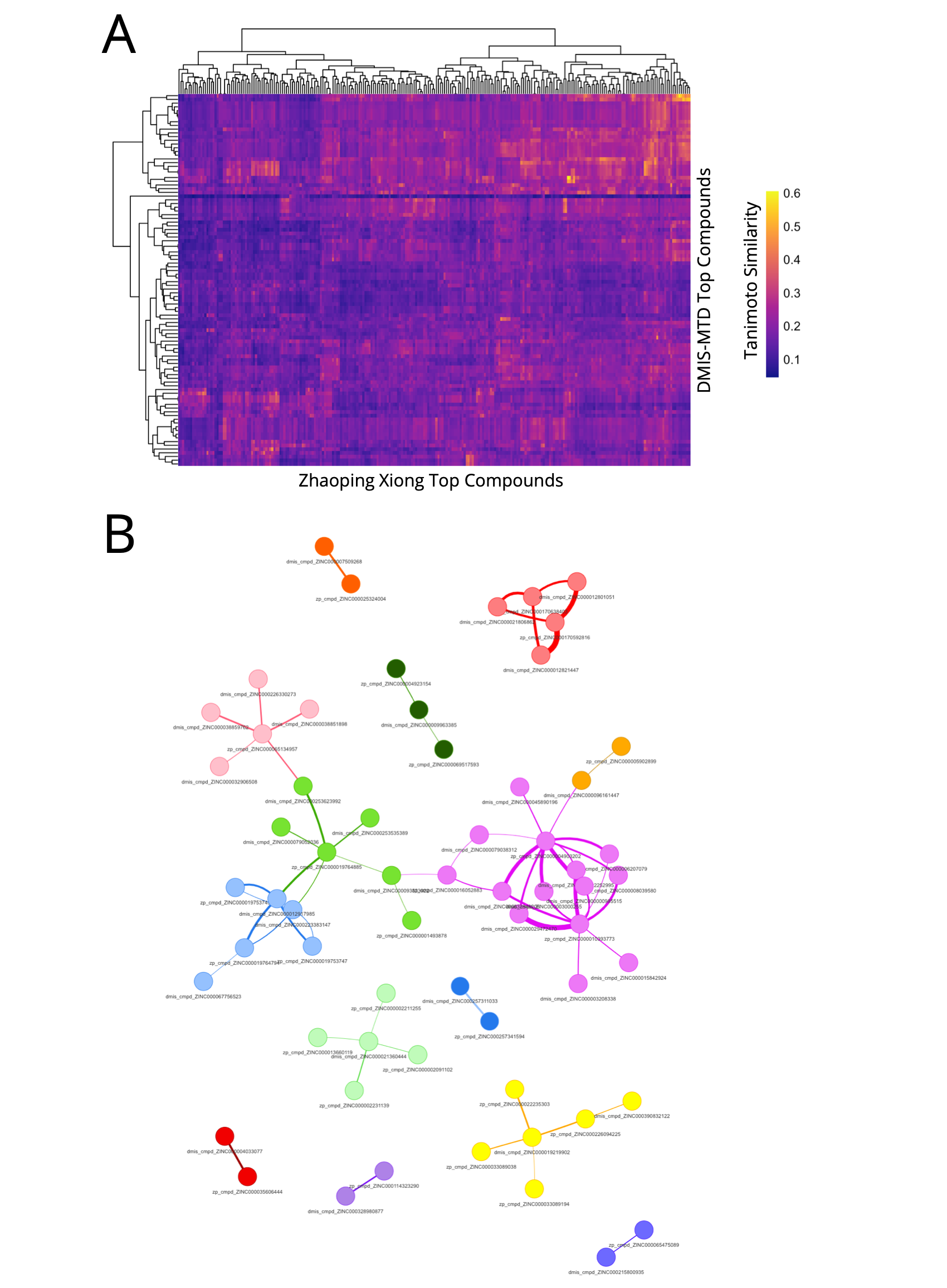

Supplement: S2 Fig — (A) Similarity heatmap of top predicted compounds for Problem 1 provided by the top-performing teams. Columns correspond to predictions from Zhaoping Xiong, while rows correspond to predictions from DMIS-MTD. The majority of compounds are relatively dissimilar from one another. (B) The similarity matrix was converted into a network, where nodes are individual predicted compounds and edges encode compound-compound Tanimoto similarities. Edge thickness represents similarity (thicker edges = greater similarity). Edges representing similarity below 0.4 were filtered out, and cluster subnetworks were identified (with each color representing an individual subnetwork). (TIFF) [file pcbi.1009302.s003.tiff]

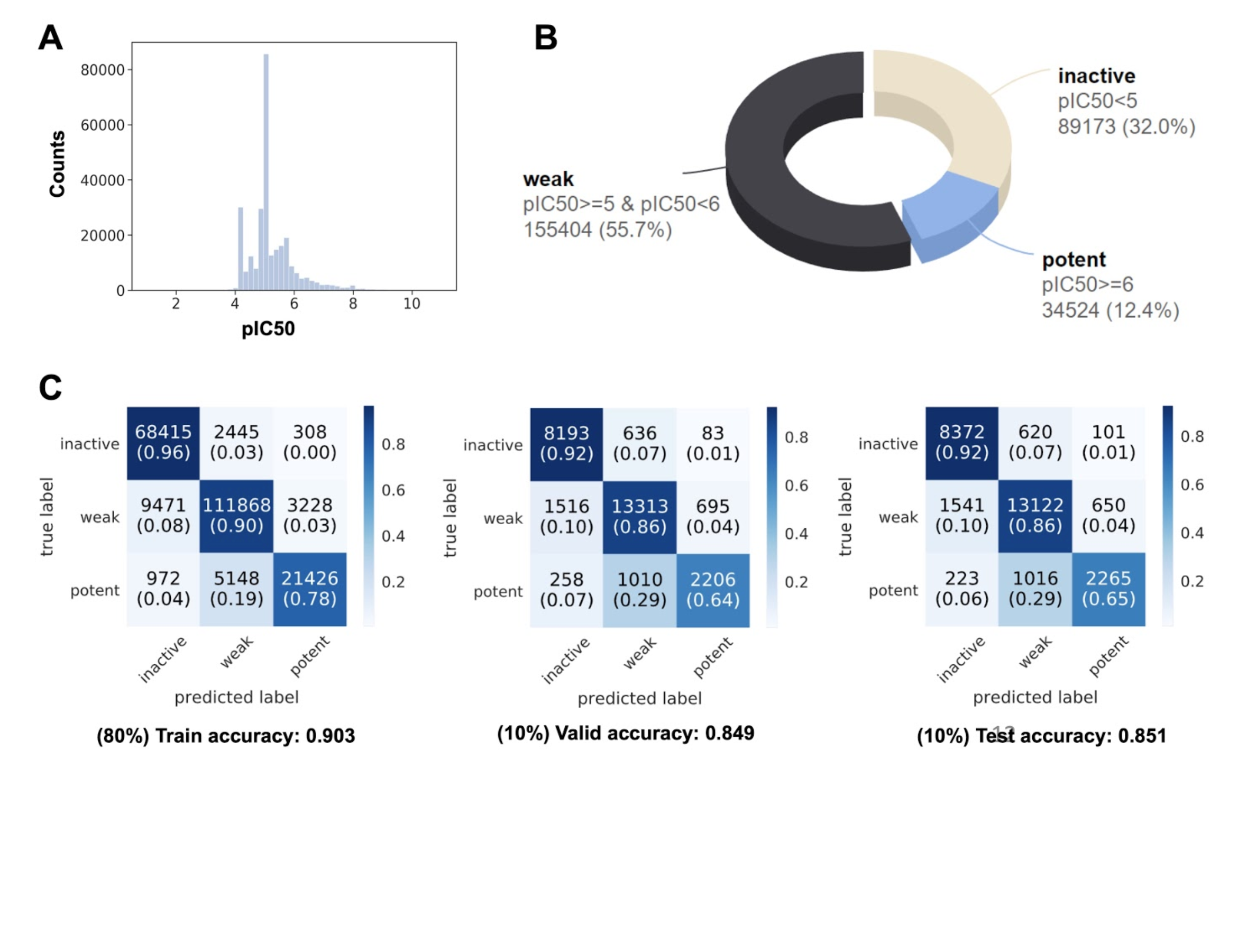

Supplement: S3 Fig — (A) The distribution of pIC50; (B) The distribution of classes; (C) The accuracy of the model across train, valid and test sets. (TIFF) [file pcbi.1009302.s004.tiff]

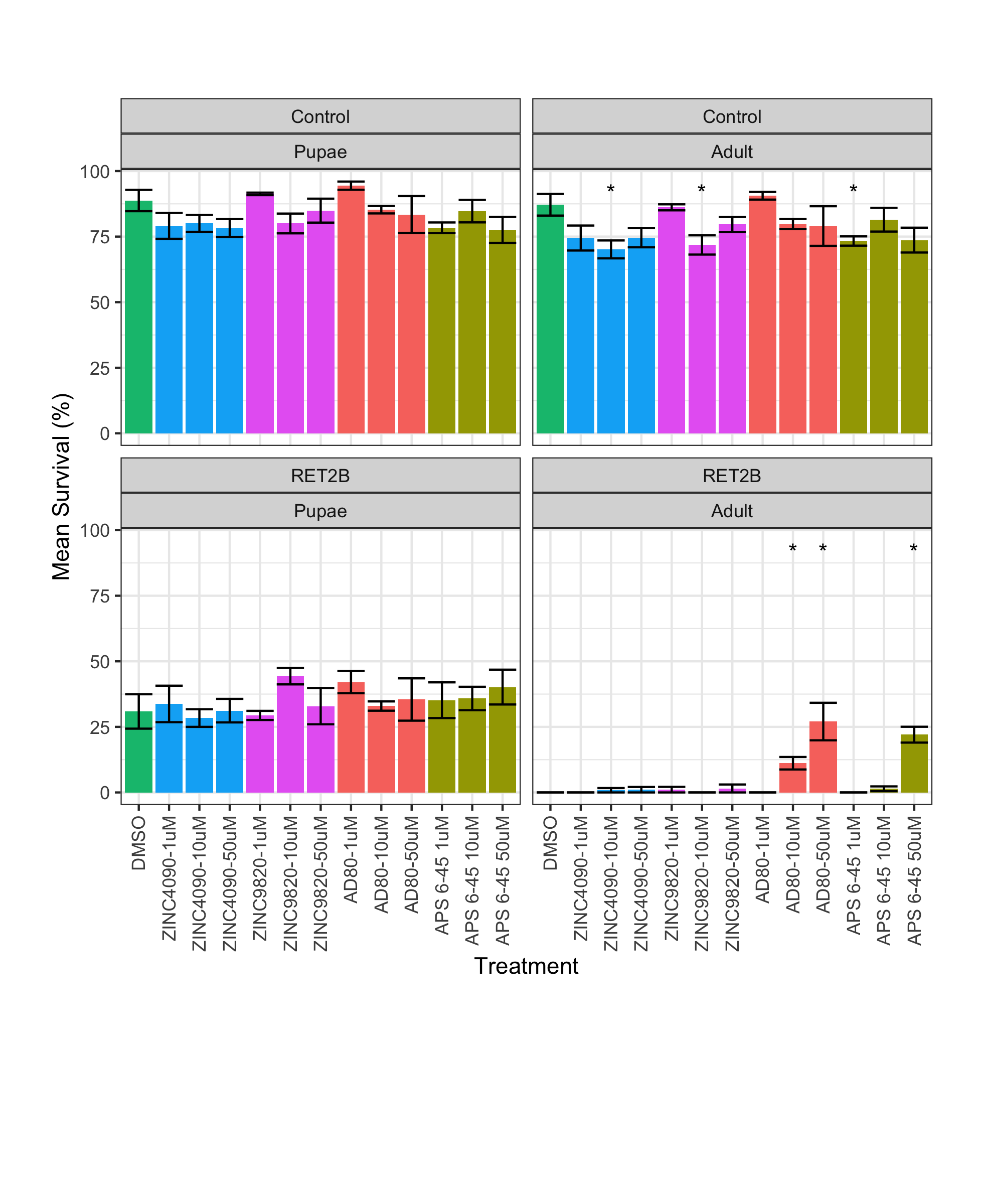

Supplement: S4 Fig — Bars show the mean percent survival and error bars show the standard deviation of four replicates per condition. A Mann-Whitney test was used to assess the significance of changes to percent survival in the varying conditions. The RET2B Adult panel (bottom right) indicates that higher concentrations of AD80 or APS6-45 significantly (p<0.05) rescue the RET2B model, while the two candidate ZINC molecules have a minimal and non statistically-significant effect on percent survival to adulthood. (TIFF) [file pcbi.1009302.s005.tiff]
