# Supplementary material for: Crowdsourced identification of multi-target kinase inhibitors for RET- and TAU- based disease: The Multi-Targeting Drug DREAM Challenge
Source: PLoS Comput Biol. 2021 Sep 14;17(9):e1009302. doi: 10.1371/journal.pcbi.1009302 (PMC8483411; doi:10.1371/journal.pcbi.1009302)
Supplement: Attachment — Submitted filename: MTD-DREAM response to reviewers.docx [file pcbi.1009302.s011.docx]

**Crowdsourced identification of multi-target kinase inhibitors for RET- and TAU- based disease: the Multi-Targeting Drug DREAM Challenge**

**Response to Reviewers**

**Reviewer #1:**

*In this manuscript, the authors reported the main results of the Multi-Targeting Drug DREAM Challenge, in developing computational methods for prediction of multi-target kinase inhibitors to treat RET-based cancer models and TAU-based neurodegenerative disease model in Drosophila. This Challenge was not organized in a very intelligent manner, and the results might not be so striking. I think the methods developed in this Challenge will be useful, however, only considering static features of proteins or small molecules might not ensure to reach a promising accuracy in reality. Since this Challenge has been finished, and indeed all participants have tried their best on method development, I think this paper can be accepted, after a minor but essential revision. I hope the authors can organize their future Challenge in a more sophisticated manner, and developed methods should touch the real fundamental problems. My major concerns are below:*

*1. The basic rationales of the Challenge were established on hypotheses. In Problem 1, the aim was prediction of small molecules that efficiently bound RET, BRAF, SRC and S6K, and didn’t bind MKNK1, TTK, ERK8, PDK1, and PAK3. In Problem 2, the aim was prediction of small molecules that efficiently bound AURKA, PAK1, FGFR1, and STK11, and didn’t bind PAK3, MAP3K7, and PIK3CA.*

*1.1) I do not think the concepts of pro-targets and anti-targets have been widely adopted by academic community, and targeting pro-targets but not anti-targets might not be the central dogma in drug design.*

Regarding the concepts of pro-targets and anti-targets, we feel these are useful terms that others are adopting: in addition to its own Wikipedia page, Google Scholar identifies “anti-target” in 1420 papers since 2017. The term “anti-target” is similar to “liability” but refers to matching liabilities to a specific assay or model. In the updated manuscript, we have included additional introductory context to highlight these differences.

*1.2) The basic hypothesis behind the Challenge is that if a small molecule can efficiently bind pro-targets but not anti-targets, corresponding diseases will be efficiently cured. I do not think this hypothesis is right. The identified small molecules might have additional side effects, and might be toxic in humans.*

In the original version of the manuscript, we were careful not to use the term “cure” to avoid readers making this leap. In the revised manuscript, we have more clearly emphasized the point that polypharmacology has risks. However, of note, the pro/anti-target data emerged from whole animal functional experiments that were defined in part as improving survival of pre-clinical models. Also of note, the purpose of the DREAM challenge was to advance the ability to achieve rational polypharmacology. The primary goal of the manuscript is not the compounds identified but the approach. Our manuscript is timely: polypharmacology is becoming increasingly embraced as a useful category of therapeutic compounds/drugs, with hundreds of reviews and commentaries in the last few years alone. Our work addresses the practical challenges of developing polypharmacological leads with reduced toxicity (anti-targets).

*1.3) The second hypothesis behind the Challenge is that experimental in Drosophila can be used in humans. I also do not agree with this point. Even experiments in mice might make nonsense in humans. Human diseases are very complicated, and I do not think fly model can be really useful. Since the Challenge has been finalized, I hope the authors can be more cautious on organizing such competition in the future. A good Challenge cannot be founded on sand piles.*

We agree that no pre-clinical platforms—including Drosophila, mouse, or human—have proven consistently predictive of success in cancer or neurodegenerative clinical trials. That is a difficult problem that is outside the scope of the design of this study.

We used Drosophila because we have the most complete genomic data from RET and Tauopathy models with flies. Again the purpose is the approach, not the compounds. We have revised portions of the Introduction to clarify this. Please also note that we previously used Drosophila to help validate the polypharmacological KI vandetanib for RET-based disease (Vidal et al, 2005), and more recently reported drug combinations to treat colorectal cancer and adenoid cystic cancer patients in a fly-to-bedside clinical trial (Bangi et al, 2019, 2021).

*2. In the biological aspect, drugs that can efficiently treat the human diseases but not one or multiple proteins will be more preferred. If a drug can indeed block some proteins but cannot efficiently treat the disease, I do not think such drugs can be useful. Thus, an additional assay should be adopted to validate whether selected drugs can efficiently inhibit RET- and TAU-based models. If the authors cannot do such experiments, they must demonstrated why selected drugs targeting some proteins but not others can be really useful in disease treatment.*

We agree that the molecules identified in this project did not have sufficient bioactivity to translate to drugs. We discuss this limitation in the Discussion and include some future directions for further research on these molecules. However, the primary goal of this project was to challenge the community to develop new strategies for identifying multi-targeting/polypharmacologic compounds; our focus is not on the clinical utility of the predicted compound, but on how well it addressed our pro/anti-target challenge.

*3. Prediction of multi-protein targeting inhibitors is an interesting topic. However, I do not think using static features derived from proteins or small molecules is enough to reach a promising accuracy. Image-base screening of small molecules using an appropriate readout might also be efficient. Thus, combination of multi-modal data types might be more useful.*

We agree that other approaches including combination approaches also have promise. We did not limit our Challenge to any particular approach. We relied on the best judgement of the participants, generally considered a strength of open competitions. We’ve added to the discussion to highlight the point raised by the reviewer that there are additional untapped methodological approaches that were not tested in this challenge.

*4. Method 1 and 2 should be described with more details. Features derived from proteins or small molecules should be listed and interpreted in an Excel table. Used parameters in neural networks should be present to enable a full reproducibility. Please give a formal names of Zhaoping Xiong’s method.*

In the updated manuscript, we refer to Method 1 (Zhaoping Xiong) as FEMTD (Fused Embedding for Multi-Targeting Drug), and we have added more detail about the model in the manuscript. To reproduce it , please refer to <https://github.com/xiongzhp/FusedEmbedding>. The final protein embeddings are available from <https://raw.githubusercontent.com/xiongzhp/FusedEmbedding/master/data/kinase_seq_embedding.csv>; we have added this link to the Code and Data Availability section of the manuscript.

Regarding ReSimNet, we now add the description of the model architecture and hyperparameters in the manuscript. We provide the input fingerprints of drugs and the output embedding vectors of the drugs in the csv formats, and now include a statement about this in the Code and Data Availability section (<https://www.synapse.org/#!Synapse:syn25702146>). However, ReSimNet takes two compounds as an input pair and the compounds are represented as 2048 bit ECFPs. Using ECFP, each substructure of the compounds is mapped into each bit of the fingerprints using a hash function, so it is challenging to get the mapped substructure from each feature dimension and interpret them. It is also difficult to interpret features derived from ReSimNet because the model generates embedding vectors of compounds in a latent space.

In addition to the details added to the manuscript, we now also add an additional note to the Methods section indicating that detailed methods write-ups are available in the Supplementary Methods document included with the manuscript.

*Taken together, I think the paper can be accepted, although the Challenge did not touch a very important problem. In the past years, a number of such Challenges have been organized. None of them really resolved any important problems.*

We thank the reviewer for their time and thoughtful insights.

**Reviewer #2:**

*The authors described the development of the Multi-Targeting Drug DREAM Challenge for the identification of multi-target kinase inhibitors. This development is necessary because multi-targeting therapeutics have key advantages over single-targeting therapeutics, but it is difficult to evaluate which molecules have desirable target profiles. The authors designed two problems and around ten teams submitted their results to this Challenge. The authors combined computational approaches and experiment validation to evaluate the submitted drugs. The models of two teams with the top scores were shared with the public through Github. This manuscript is well-structured. I recommend it for publication.*

We thank the reviewer for their time and feedback.
